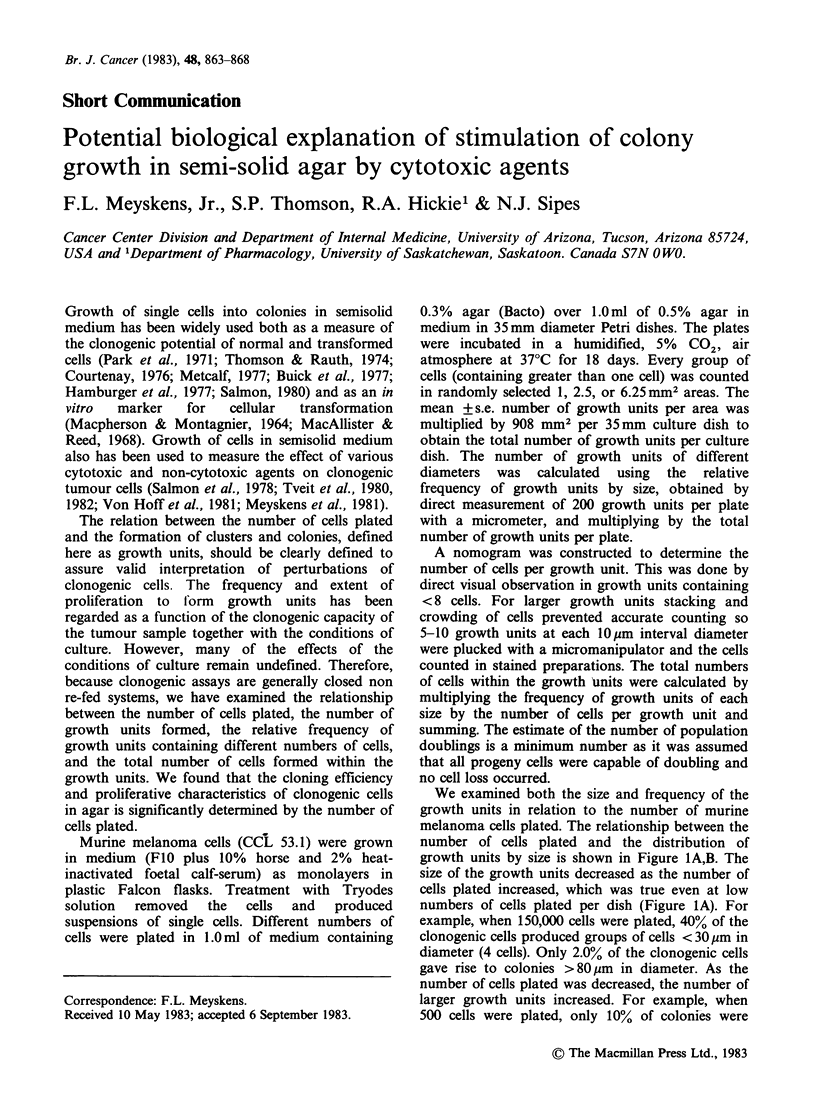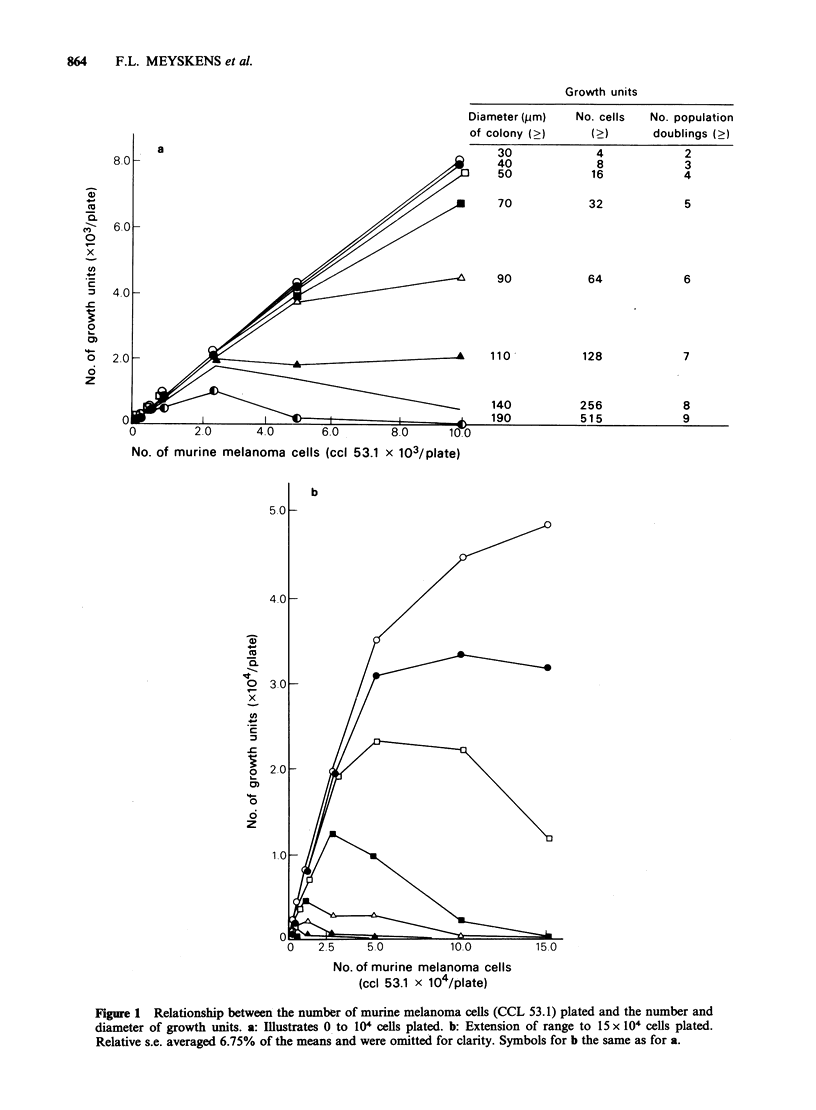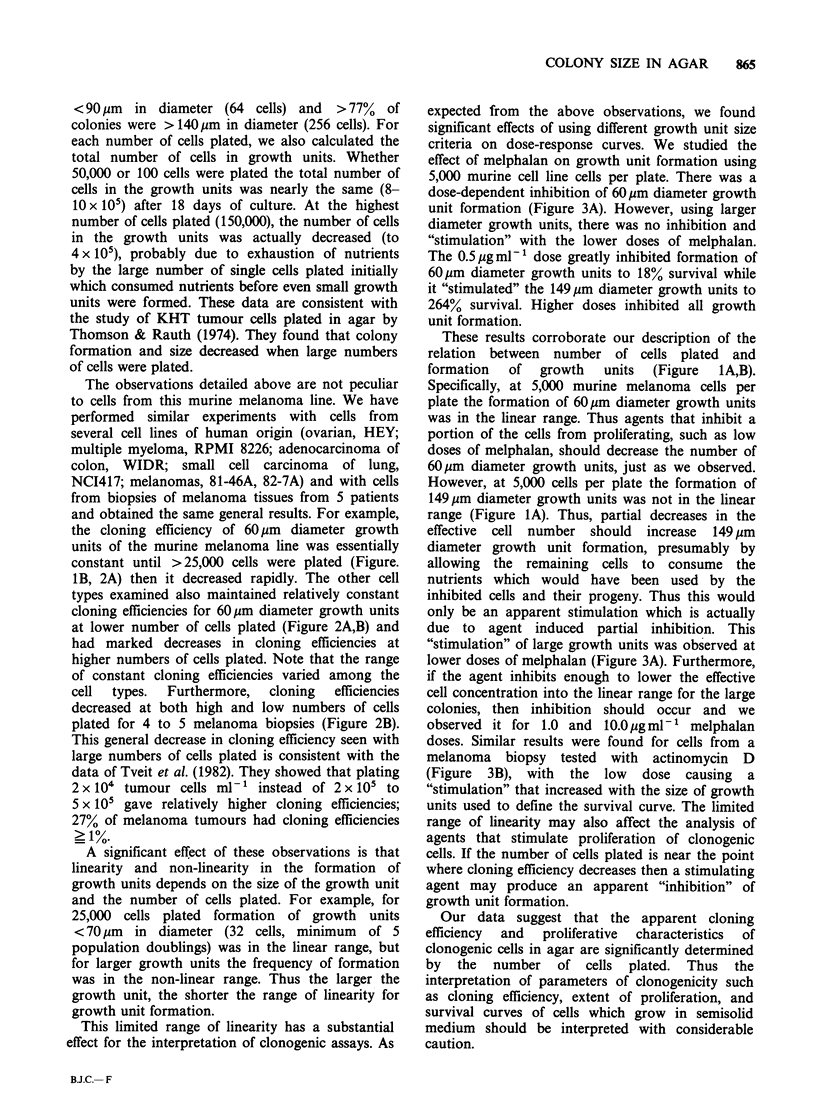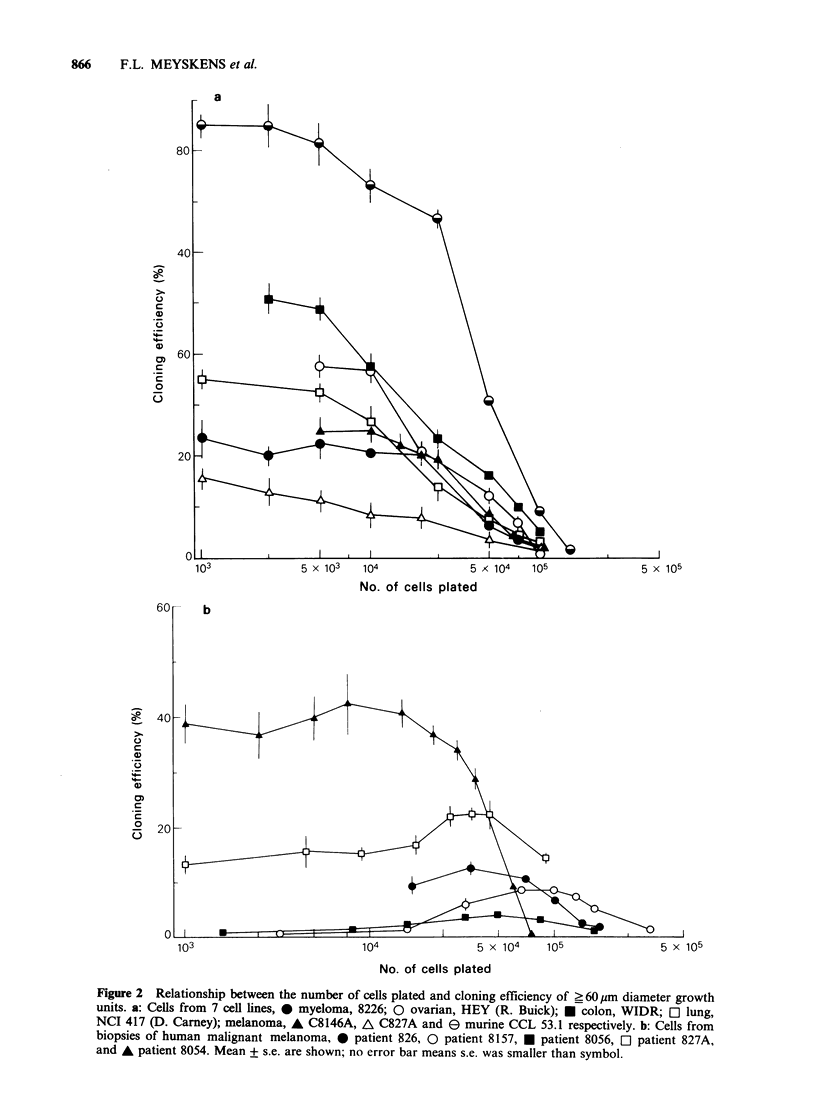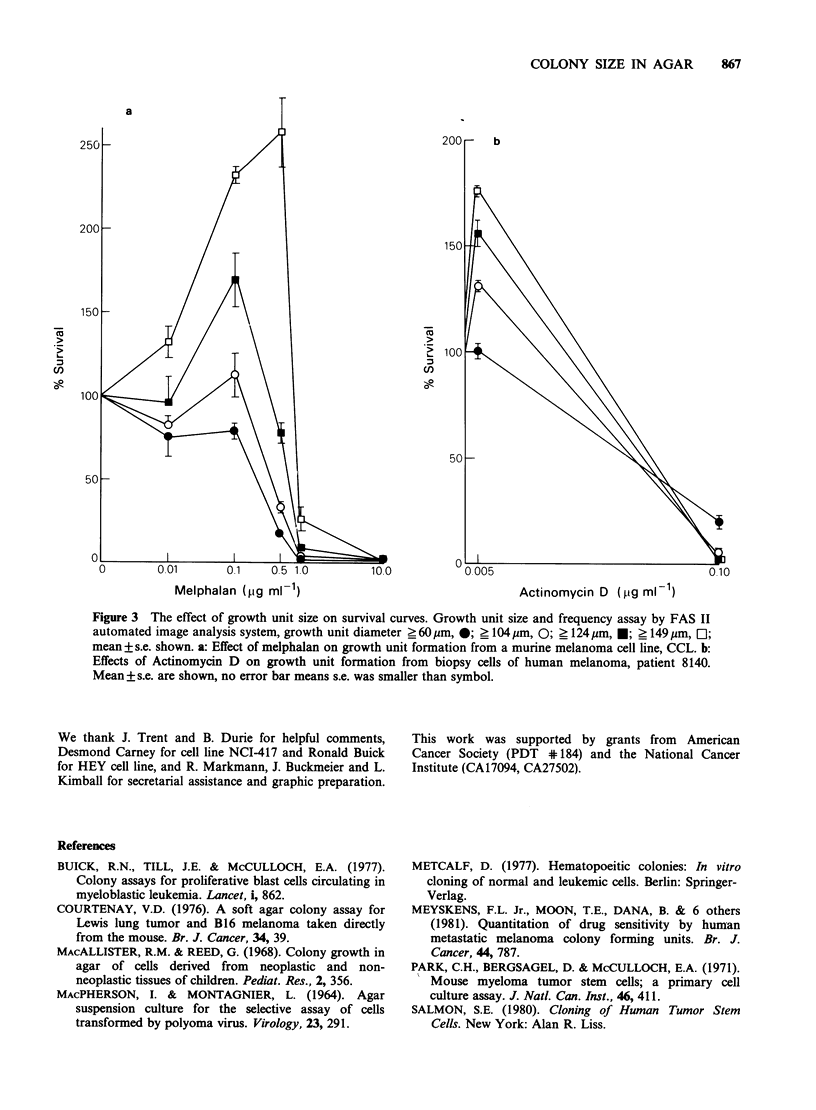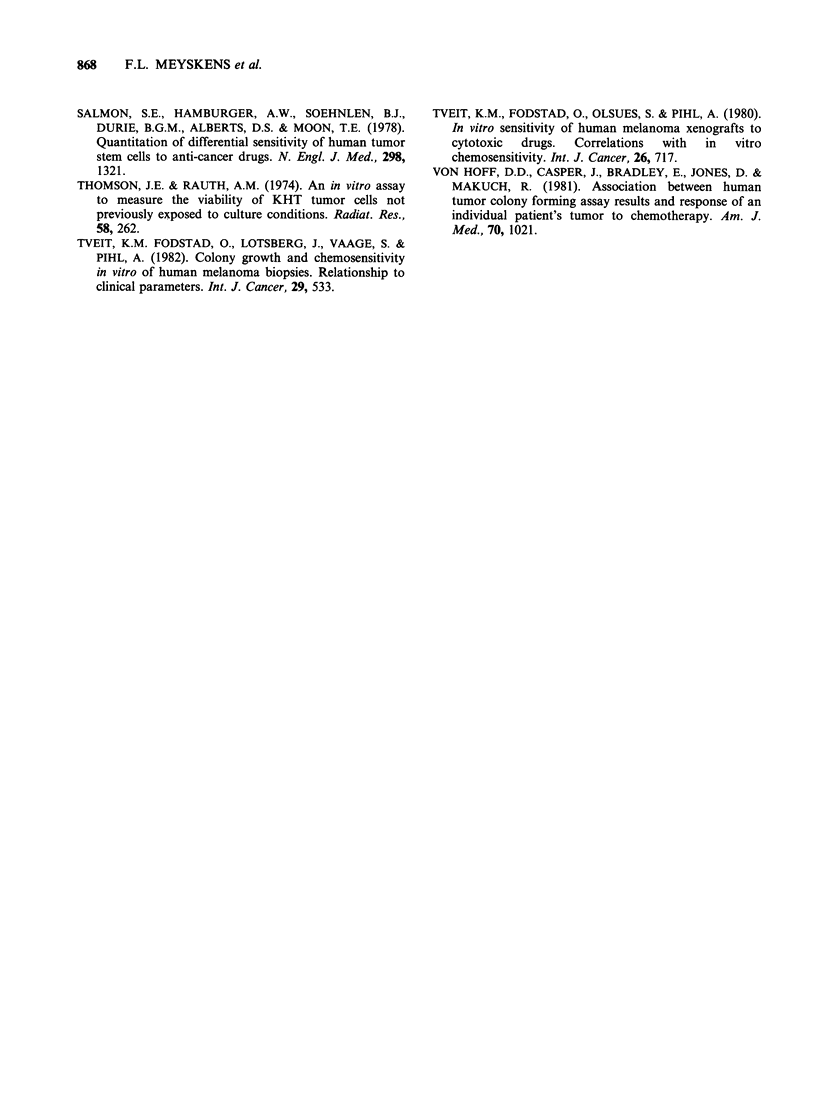# Potential biological explanation of stimulation of colony growth in semi-solid agar by cytotoxic agents.

**DOI:** 10.1038/bjc.1983.279

**Published:** 1983-12

**Authors:** F. L. Meyskens, S. P. Thomson, R. A. Hickie, N. J. Sipes


*Br. J. Cancer* (1983), **48**, 863–868

**Short Communication**

### Potential biological explanation of stimulation of colony growth in semi-solid agar by cytotoxic agents

F.L. Meyskens, Jr., S.P. Thomson, R.A. Hickie[1] & N.J. Sipes

*Cancer Center Division and Department of Internal Medicine, University of Arizona, Tucson, Arizona 85724, USA and [1]Department of Pharmacology, University of Saskatchewan, Saskatoon. Canada S7N 0W0.*

Growth of single cells into colonies in semisolid medium has been widely used both as a measure of the clonogenic potential of normal and transformed cells (Park *et al.*, 1971; Thomson & Rauth, 1974; Courtenay, 1976; Metcalf, 1977; Buick *et al.*, 1977; Hamburger *et al.*, 1977; Salmon, 1980) and as an *in vitro* marker for cellular transformation (Macpherson & Montagnier, 1964; MacAllister & Reed, 1968). Growth of cells in semisolid medium also has been used to measure the effect of various cytotoxic and non-cytotoxic agents on clonogenic tumour cells (Salmon *et al.*, 1978; Tveit *et al.*, 1980, 1982; Von Hoff *et al.*, 1981; Meyskens *et al.*, 1981).

The relation between the number of cells plated and the formation of clusters and colonies, defined here as growth units, should be clearly defined to assure valid interpretation of perturbations of clonogenic cells. The frequency and extent of proliferation to form growth units has been regarded as a function of the clonogenic capacity of the tumour sample together with the conditions of culture. However, many of the effects of the conditions of culture remain undefined. Therefore, because clonogenic assays are generally closed non re-fed systems, we have examined the relationship between the number of cells plated, the number of growth units formed, the relative frequency of growth units containing different numbers of cells, and the total number of cells formed within the growth units. We found that the cloning efficiency and proliferative characteristics of clonogenic cells in agar is significantly determined by the number of cells plated.

Murine melanoma cells (CCL 53.1) were grown in medium (F10 plus 10% horse and 2% heat-inactivated foetal calf-serum) as monolayers in plastic Falcon flasks. Treatment with Tryodes solution removed the cells and produced suspensions of single cells. Different numbers of cells were plated in 1.0 ml of medium containing 0.3% agar (Bacto) over 1.0 ml of 0.5% agar in medium in 35 mm diameter Petri dishes. The plates were incubated in a humidified, 5% $CO_2$, air atmosphere at 37°C for 18 days. Every group of cells (containing greater than one cell) was counted in randomly selected 1, 2.5, or 6.25 mm² areas. The mean ±s.e. number of growth units per area was multiplied by 908 mm² per 35 mm culture dish to obtain the total number of growth units per culture dish. The number of growth units of different diameters was calculated using the relative frequency of growth units by size, obtained by direct measurement of 200 growth units per plate with a micrometer, and multiplying by the total number of growth units per plate.

A nomogram was constructed to determine the number of cells per growth unit. This was done by direct visual observation in growth units containing <8 cells. For larger growth units stacking and crowding of cells prevented accurate counting so 5–10 growth units at each 10 $\mu$m interval diameter were plucked with a micromanipulator and the cells counted in stained preparations. The total numbers of cells within the growth units were calculated by multiplying the frequency of growth units of each size by the number of cells per growth unit and summing. The estimate of the number of population doublings is a minimum number as it was assumed that all progeny cells were capable of doubling and no cell loss occurred.

We examined both the size and frequency of the growth units in relation to the number of murine melanoma cells plated. The relationship between the number of cells plated and the distribution of growth units by size is shown in Figure 1A,B. The size of the growth units decreased as the number of cells plated increased, which was true even at low numbers of cells plated per dish (Figure 1A). For example, when 150,000 cells were plated, 40% of the clonogenic cells produced groups of cells <30 $\mu$m in diameter (4 cells). Only 2.0% of the clonogenic cells gave rise to colonies >80 $\mu$m in diameter. As the number of cells plated was decreased, the number of larger growth units increased. For example, when 500 cells were plated, only 10% of colonies were

Correspondence: F.L. Meyskens.
Received 10 May 1983; accepted 6 September 1983.

© The Macmillan Press Ltd., 1983

| Growth units | | |
|---|---|---|
| Diameter (μm) of colony (≥) | No. cells (≥) | No. population doublings (≥) |
| 30 | 4 | 2 |
| 40 | 8 | 3 |
| 50 | 16 | 4 |
| 70 | 32 | 5 |
| 90 | 64 | 6 |
| 110 | 128 | 7 |
| 140 | 256 | 8 |
| 190 | 515 | 9 |

**Figure 1** Relationship between the number of murine melanoma cells (CCL 53.1) plated and the number and diameter of growth units. **a**: Illustrates 0 to $10^4$ cells plated. **b**: Extension of range to $15 \times 10^4$ cells plated. Relative s.e. averaged 6.75% of the means and were omitted for clarity. Symbols for **b** the same as for **a**.

$<90\,\mu m$ in diameter (64 cells) and $>77\%$ of colonies were $>140\,\mu m$ in diameter (256 cells). For each number of cells plated, we also calculated the total number of cells in growth units. Whether 50,000 or 100 cells were plated the total number of cells in the growth units was nearly the same (8–$10\times10^5$) after 18 days of culture. At the highest number of cells plated (150,000), the number of cells in the growth units was actually decreased (to $4\times10^5$), probably due to exhaustion of nutrients by the large number of single cells plated initially which consumed nutrients before even small growth units were formed. These data are consistent with the study of KHT tumour cells plated in agar by Thomson & Rauth (1974). They found that colony formation and size decreased when large numbers of cells were plated.

The observations detailed above are not peculiar to cells from this murine melanoma line. We have performed similar experiments with cells from several cell lines of human origin (ovarian, HEY; multiple myeloma, RPMI 8226; adenocarcinoma of colon, WIDR; small cell carcinoma of lung, NCI417; melanomas, 81-46A, 82-7A) and with cells from biopsies of melanoma tissues from 5 patients and obtained the same general results. For example, the cloning efficiency of $60\,\mu m$ diameter growth units of the murine melanoma line was essentially constant until $>25{,}000$ cells were plated (Figure. 1B, 2A) then it decreased rapidly. The other cell types examined also maintained relatively constant cloning efficiencies for $60\,\mu m$ diameter growth units at lower number of cells plated (Figure 2A,B) and had marked decreases in cloning efficiencies at higher numbers of cells plated. Note that the range of constant cloning efficiencies varied among the cell types. Furthermore, cloning efficiencies decreased at both high and low numbers of cells plated for 4 to 5 melanoma biopsies (Figure 2B). This general decrease in cloning efficiency seen with large numbers of cells plated is consistent with the data of Tveit *et al.* (1982). They showed that plating $2\times10^4$ tumour cells $ml^{-1}$ instead of $2\times10^5$ to $5\times10^5$ gave relatively higher cloning efficiencies; 27% of melanoma tumours had cloning efficiencies $\geqq 1\%$.

A significant effect of these observations is that linearity and non-linearity in the formation of growth units depends on the size of the growth unit and the number of cells plated. For example, for 25,000 cells plated formation of growth units $<70\,\mu m$ in diameter (32 cells, minimum of 5 population doublings) was in the linear range, but for larger growth units the frequency of formation was in the non-linear range. Thus the larger the growth unit, the shorter the range of linearity for growth unit formation.

This limited range of linearity has a substantial effect for the interpretation of clonogenic assays. As expected from the above observations, we found significant effects of using different growth unit size criteria on dose-response curves. We studied the effect of melphalan on growth unit formation using 5,000 murine cell line cells per plate. There was a dose-dependent inhibition of $60\,\mu m$ diameter growth unit formation (Figure 3A). However, using larger diameter growth units, there was no inhibition and "stimulation" with the lower doses of melphalan. The $0.5\,\mu g\,ml^{-1}$ dose greatly inhibited formation of $60\,\mu m$ diameter growth units to 18% survival while it "stimulated" the $149\,\mu m$ diameter growth units to 264% survival. Higher doses inhibited all growth unit formation.

These results corroborate our description of the relation between number of cells plated and formation of growth units (Figure 1A,B). Specifically, at 5,000 murine melanoma cells per plate the formation of $60\,\mu m$ diameter growth units was in the linear range. Thus agents that inhibit a portion of the cells from proliferating, such as low doses of melphalan, should decrease the number of $60\,\mu m$ diameter growth units, just as we observed. However, at 5,000 cells per plate the formation of $149\,\mu m$ diameter growth units was not in the linear range (Figure 1A). Thus, partial decreases in the effective cell number should increase $149\,\mu m$ diameter growth unit formation, presumably by allowing the remaining cells to consume the nutrients which would have been used by the inhibited cells and their progeny. Thus this would only be an apparent stimulation which is actually due to agent induced partial inhibition. This "stimulation" of large growth units was observed at lower doses of melphalan (Figure 3A). Furthermore, if the agent inhibits enough to lower the effective cell concentration into the linear range for the large colonies, then inhibition should occur and we observed it for 1.0 and $10.0\,\mu g\,ml^{-1}$ melphalan doses. Similar results were found for cells from a melanoma biopsy tested with actinomycin D (Figure 3B), with the low dose causing a "stimulation" that increased with the size of growth units used to define the survival curve. The limited range of linearity may also affect the analysis of agents that stimulate proliferation of clonogenic cells. If the number of cells plated is near the point where cloning efficiency decreases then a stimulating agent may produce an apparent "inhibition" of growth unit formation.

Our data suggest that the apparent cloning efficiency and proliferative characteristics of clonogenic cells in agar are significantly determined by the number of cells plated. Thus the interpretation of parameters of clonogenicity such as cloning efficiency, extent of proliferation, and survival curves of cells which grow in semisolid medium should be interpreted with considerable caution.

**Figure 2** Relationship between the number of cells plated and cloning efficiency of $\geq 60\,\mu m$ diameter growth units. **a**: Cells from 7 cell lines, ● myeloma, 8226; ○ ovarian, HEY (R. Buick); ■ colon, WIDR; □ lung, NCI 417 (D. Carney); melanoma, ▲ C8146A, △ C827A and ⊖ murine CCL 53.1 respectively. **b**: Cells from biopsies of human malignant melanoma, ● patient 826, ○ patient 8157, ■ patient 8056, □ patient 827A, and ▲ patient 8054. Mean ± s.e. are shown; no error bar means s.e. was smaller than symbol.

**Figure 3** The effect of growth unit size on survival curves. Growth unit size and frequency assay by FAS II automated image analysis system, growth unit diameter ≧60 μm, ●; ≧104 μm, ○; ≧124 μm, ■; ≧149 μm, □; mean ± s.e. shown. **a**: Effect of melphalan on growth unit formation from a murine melanoma cell line, CCL. **b**: Effects of Actinomycin D on growth unit formation from biopsy cells of human melanoma, patient 8140. Mean ± s.e. are shown, no error bar means s.e. was smaller than symbol.

We thank J. Trent and B. Durie for helpful comments, Desmond Carney for cell line NCI-417 and Ronald Buick for HEY cell line, and R. Markmann, J. Buckmeier and L. Kimball for secretarial assistance and graphic preparation.

This work was supported by grants from American Cancer Society (PDT #184) and the National Cancer Institute (CA17094, CA27502).

### References

BUICK, R.N., TILL, J.E. & McCULLOCH, E.A. (1977). Colony assays for proliferative blast cells circulating in myeloblastic leukemia. *Lancet*, **i**, 862.

COURTENAY, V.D. (1976). A soft agar colony assay for Lewis lung tumor and B16 melanoma taken directly from the mouse. *Br. J. Cancer*, **34**, 39.

MacALLISTER, R.M. & REED, G. (1968). Colony growth in agar of cells derived from neoplastic and non-neoplastic tissues of children. *Pediat. Res.*, **2**, 356.

MacPHERSON, I. & MONTAGNIER, L. (1964). Agar suspension culture for the selective assay of cells transformed by polyoma virus. *Virology*, **23**, 291.

METCALF, D. (1977). Hematopoeitic colonies: *In vitro* cloning of normal and leukemic cells. Berlin: Springer-Verlag.

MEYSKENS, F.L. Jr., MOON, T.E., DANA, B. & 6 others (1981). Quantitation of drug sensitivity by human metastatic melanoma colony forming units. *Br. J. Cancer*, **44**, 787.

PARK, C.H., BERGSAGEL, D. & McCULLOCH, E.A. (1971). Mouse myeloma tumor stem cells; a primary cell culture assay. *J. Natl. Can. Inst.*, **46**, 411.

SALMON, S.E. (1980). *Cloning of Human Tumor Stem Cells*. New York: Alan R. Liss.

SALMON, S.E., HAMBURGER, A.W., SOEHNLEN, B.J., DURIE, B.G.M., ALBERTS, D.S. & MOON, T.E. (1978). Quantitation of differential sensitivity of human tumor stem cells to anti-cancer drugs. *N. Engl. J. Med.*, **298**, 1321.

THOMSON, J.E. & RAUTH, A.M. (1974). An *in vitro* assay to measure the viability of KHT tumor cells not previously exposed to culture conditions. *Radiat. Res.*, **58**, 262.

TVEIT, K.M. FODSTAD, O., LOTSBERG, J., VAAGE, S. & PIHL, A. (1982). Colony growth and chemosensitivity *in vitro* of human melanoma biopsies. Relationship to clinical parameters. *Int. J. Cancer*, **29**, 533.

TVEIT, K.M., FODSTAD, O., OLSUES, S. & PIHL, A. (1980). *In vitro* sensitivity of human melanoma xenografts to cytotoxic drugs. Correlations with in vitro chemosensitivity. *Int. J. Cancer*, **26**, 717.

VON HOFF, D.D., CASPER, J., BRADLEY, E., JONES, D. & MAKUCH, R. (1981). Association between human tumor colony forming assay results and response of an individual patient's tumor to chemotherapy. *Am. J. Med.*, **70**, 1021.